# Comparison of Cerebrospinal Fluid Amyloidogenic Nanoplaques With Core Biomarkers of Alzheimer’s Disease

**DOI:** 10.3389/fnagi.2020.608628

**Published:** 2021-01-08

**Authors:** Mari Aksnes, Ann Tiiman, Trine Holt Edwin, Lars Terenius, Nenad Bogdanović, Vladana Vukojević, Anne-Brita Knapskog

**Affiliations:** ^1^Department of Geriatric Medicine, Institute of Clinical Medicine, Faculty of Medicine, University of Oslo, Oslo, Norway; ^2^Department of Clinical Neurosciences (CNS), Center for Molecular Medicine CMM L8:01, Karolinska Institutet, Stockholm, Sweden; ^3^Institute of Health and Society, Faculty of Medicine, University of Oslo, Oslo, Norway; ^4^Department of Geriatric Medicine, The Memory Clinic, Oslo University Hospital, Oslo, Norway; ^5^Norwegian National Advisory Unit on Ageing and Health, Vestfold Hospital Trust, Oslo, Norway; ^6^Department of Neurobiology, Care Science and Society (NVS), Division of Clinical Geriatrics, Karolinska Institutet, Huddinge, Sweden

**Keywords:** Alzheimer disease, amyloid, amyloid beta-peptides, amyloidogenic proteins, biomarkers, cerebrospinal fluid, fluorescence spectrometry, thioflavin T

## Abstract

Accurate biomarkers of Alzheimer’s disease (AD) are essential for early diagnosis and intervention. Available biomarkers are not sufficient to permit the monitoring of AD progression over time, and additional biomarkers are required. Measures of aggregated amyloid-β (Aβ) could be useful biomarkers for AD. Here, we investigate whether levels of Thioflavin-T (ThT) positive amyloid aggregates, i.e., nanoplaques, in cerebrospinal fluid (CSF) could serve as useful biomarkers for AD. One-hundred and eighteen memory clinic patients were AT(N) classified, and CSF nanoplaque concentrations were compared between patients on the “Alzheimer’s continuum” (A+ patients) and patients with “Normal AD biomarkers” or “Non-AD pathologic change” (A− patients). CSF nanoplaque concentrations and sizes were quantified using the novel ThT-Fluorescence Correlation Spectroscopy (ThT-FCS) assay, and core biomarkers (Aβ_42_, total tau and phosphorylated tau) were determined by enzyme-linked immunosorbent assays. We investigated the association between nanoplaque concentrations and core biomarkers, and the diagnostic value of nanoplaque levels. Nanoplaque levels were increased in A+ patients compared to A− patients. Nanoplaque concentrations were negatively associated with Aβ_42_, but not related to total tau or phosphorylated tau measures. Quantification of nanoplaques did not improve the classification of patients on the Alzheimer’s continuum compared to the core biomarkers alone. Dynamic changes in nanoplaques concentration and size throughout AD stages should be explored in longitudinal studies.

## Introduction

There is currently no cure for Alzheimer’s disease (AD), the leading cause of dementia worldwide. While detailed cellular and molecular mechanisms underlying AD remain elusive, the amyloid cascade hypothesis postulates that AD is caused by the accumulation of aggregated amyloid-β (Aβ) peptides (Hardy and Higgins, 1992; Selkoe and Hardy, 2016). It is hypothesized that these aggregates induce phosphorylation and aggregation of the tau protein into neurofibrillary tangles, before eventually forming amyloid plaques (Selkoe and Hardy, 2016). Indeed, Aβ pathology has been demonstrated to induce tau phosphorylation and aggregation in human neural cell culture models of AD (Choi et al., 2014; Kwak et al., 2020). It has been established that amyloid pathology develops several years before the first clinical symptoms of dementia arise (Jack et al., 2013). This preclinical AD stage is of great interest because it is believed to be the optimal period for pharmacological intervention (Blennow et al., 2015). Consequently, biomarkers indicative of the underlying pathological processes could be indispensable for identifying patients for clinical trials (Aluise et al., 2008), and are increasingly incorporated in AD diagnostic procedures (McKhann et al., 2011).

Several imaging and cerebrospinal fluid (CSF) biomarkers for AD have already been identified (Perrin et al., 2009; Jack et al., 2016). In CSF, three core biomarkers have been validated as markers of AD neuropathology (Blennow et al., 2015): low levels of Aβ_42_ are indicative of brain amyloid pathology, elevated levels of phosphorylated tau (P-tau) signify tau pathology, and elevated levels of total tau reflect neurodegeneration (T-tau). These markers, and their neuroimaging counterparts, each represent a biomarker group in the NIA-AA research framework (Jack et al., 2018). In this framework, markers are classified in an AT(N) system where “A” reflects markers of Aβ deposition, “T” reflects markers of pathologic tau, and “N” reflects markers of neurodegeneration. Each biomarker group can be defined as normal or abnormal (+/−), resulting in eight possible biomarker profiles.

While the core AD biomarkers accurately reflect AD neuropathology, they have several limitations. First, the core biomarkers do not inform on disease progression, because they are relatively stable in the clinical stages of AD (Dhiman et al., 2019). Second, the core biomarkers have limited use as primary endpoints in clinical trials, as they do not correlate well with cognitive function (Zhou et al., 2009; Williams et al., 2011; Radanovic et al., 2019), and changes in core biomarker levels do not correlate with cognitive change (Toledo et al., 2013). Accordingly, the AT(N) biomarker profiles have recently been found to have restricted use for the prediction of AD progression rates (Wattmo et al., 2020). Finally, the core biomarkers do not differentiate AD from other causes of dementia with sufficient accuracy (Blennow et al., 2012). There is therefore a need for supplemental biomarkers that permit monitoring of AD progression over time and reflect the response (if any) to therapeutic interventions.

Measures of aggregated Aβ are promising candidate biomarkers. Increased levels of monomeric Aβ do not cause neurodegeneration; the self-association of Aβ into aggregates is necessary (Funke et al., 2009). It is theorized that the lowering of CSF monomeric Aβ_42_ in preclinical AD is due to increased retention of Aβ in neurite plaques in the brain. In line with this, low CSF Aβ_42_ is found to correlate with increased brain uptake of amyloid ligands on amyloid-positron emission tomography (amyloid-PET; Forsberg et al., 2008; Müller et al., 2019). Consequently, measures of amyloid aggregates would be an early biomarker and reflect ongoing pathology (Holtta et al., 2013). As such, they could be useful markers of the disease process and potential treatment responses. The novel Thioflavin-T Fluorescence Correlation Spectroscopy (ThT-FCS) assay uses ThT to label and quantify structured amyloidogenic aggregates with high specificity. Recently, this method has been used to identify amyloid aggregates with single-molecule sensitivity; both synthetic aggregates *in vitro* (Tiiman et al., 2015) and aggregates in blood serum (Tiiman et al., 2019) and CSF (Aksnes et al., 2020).

In this article, we investigate the relationship between core CSF biomarkers and average nanoplaque concentrations and sizes determined using ThT-FCS, in memory clinic patients with different AT(N)-profiles.

## Materials and Methods

### Study Cohort

Patients from the Norwegian Registry of Persons Assessed for Cognitive Symptoms (NorCog), who had been referred for cognitive complaints to the Oslo University Hospital Memory Clinic, were included in the study. All patients were deemed to have sufficient cognitive capacity to provide informed consent at the time of inclusion, and all patients signed written informed consent. Patients were included between June 2014 and November 2018 and underwent CSF sampling as part of the diagnostic procedure. Clinical data were extracted from NorCog, or patient medical records if missing from the registry.

#### Clinical Assessment

The clinical assessment followed a standardized research protocol (Braekhus et al., 2011). Patients and their caregivers were interviewed about symptoms, medication, and medical history. The cognitive assessment included the Mini-Mental State Examination (MMSE; Folstein et al., 1975) and further cognitive tests. The extent of cognitive and functional impairment on the Clinical Dementia Rating scale (CDR; Hughes et al., 1982) was scored *post hoc* using all available information, by THE and ABK, experienced CDR raters. The physical assessment included blood and CSF sampling for all patients (*N* = 118), neuroimaging with magnetic resonance imaging (MRI, *N* = 114), and for a subset of patients, ^18^F-FDG PET (*N* = 71) and/or ^18^F-Flutemetamol-PET (*N* = 54). Atrophy on MRI was determined according to the cut-offs published by Ferreira and colleagues (Ferreira et al., 2015), ^18^F-Flutemetamol PET scans were visually classified as positive or negative in line with a validated reader program (Buckley et al., 2017), and ^18^F-FDG PET scans were evaluated according to the European Association of Nuclear Medicine Guidelines (Varrone et al., 2009).

Research diagnoses were determined retrospectively by THE and ABK, experienced memory clinic physicians. Cognitive impairment was staged as subjective cognitive decline (SCD), mild cognitive impairment (MCI) or dementia according to the SCD Initiative- (Jessen et al., 2014) and the National Institute of Aging and the Alzheimer’s Association (NIA-AA)-criteria (Albert et al., 2011; McKhann et al., 2011), respectively. Clinical diagnoses were grouped as Alzheimer’s clinical syndrome (corresponding to probable/possible AD according to the 2011 NIA-AA-criteria) or clinically non-AD (including SCD, vascular dementia (Sachdev et al., 2014), frontotemporal dementia (Rascovsky et al., 2011) and uncategorized cases). Patients with dementia with Lewy bodies, a non-AD amyloidogenic disorder, were excluded.

#### AT(N)-Classification

Patients were classified into eight AT(N) profiles based on all available biomarkers: for A, CSF Aβ_42_ levels and/or ^18^F-Flutemetamol classification; for T, CSF P-tau; for N, CSF T-tau, ^18^F-FDG and/or MRI results. In the case of discrepancies between the different biomarkers in one group, e.g., T-tau levels below the cut-off, but evidence of neuronal injury on MRI, the patient was classified as positive. The resulting eight AT(N) profiles were grouped into three biomarker categories: “Normal AD biomarkers” [A-T−(N−)], “Alzheimer’s continuum” [A + T−(N−), A + T + (N−), A + T + (N+), A + T−(N+)], or “Non-AD pathologic change” [A−T + (N−), A−T−(N+), A−T + (N+)]. Also, the profiles were divided into two groups (A +/A−) based on amyloid status only.

### Analysis of CSF Core Biomarkers

Lumbar puncture with subsequent measurement of CSF core biomarkers (Aβ_42_, T-tau, and P-tau) was performed for all patients. Core biomarkers were analyzed with Innotest Kit enzyme-linked immunosorbent assays (Innogenetics, Ghent, Belgium) at the Department for Interdisciplinary Laboratory Medicine and Medical Biochemistry at Akershus University Hospital, Norway, which participates in the Alzheimer’s Association QC program for CSF biomarkers (Mattsson et al., 2011). Laboratory recommended cut-off values for a normal test were applied (Kalheim et al., 2018): Aβ_42_ above 700 pg/ml; P-tau below 80 pg/ml; and T-tau below 300 pg/ml for patients younger than 50 years, below 450 pg/ml for patients aged 50–69 years, and below 500 pg/ml for patients older than 70 years.

### Analysis of CSF Nanoplaque Concentrations by ThT-FCS

CSF samples from all included patients were obtained from the NorCog biobank. Fluorescence intensity fluctuations were recorded using an individually modified ConfoCor3 system (Carl Zeiss, Jena, Germany; Vukojević et al., 2008). The procedure for ThT-FCS analysis is described in detail in Tiiman et al. (2019).

In brief, 1.6 μl of 2.5 mM ThT in deionized water was added to 200 μl CSF. Fluorescence intensity fluctuations were recorded in duplicates. The signal was collected for 3,000 s (30 series of 10 × 10 s measurements). Bursts in fluorescence intensity reflecting the passage of ThT-positive aggregates, i.e., nanoplaques, through the observation volume element were detected by automated fluorescence intensity fluctuation analysis. An increase in fluorescence intensity by a value that is more than five times larger than the standard deviation of the whole time series was denoted as a “single event.” The frequency of single events (*f*SEO), i.e., the total number of single events per hour, is a direct measure of the concentration of nanoplaques.

### Analysis of CSF Nanoplaque Sizes

To analyze nanoplaque size, temporal autocorrelation curves (tACCs) were derived by temporal autocorrelation analysis of time series where single events were noted. The tACCs were normalized to the same amplitude, *G*n(t) = 1, at time τ = 10 μs, and averaged across A− and A+ patients, and across “Normal AD biomarkers,” “Alzheimer’s continuum” and “Non-AD pathologic change” biomarker categories. Because only a small number of single events were observed for each individual, nanoplaque size could not be determined at the individual level. The averaged tACCs were evaluated using the Maximum Entropy Method for FCS (MEMFCS; Sengupta et al., 2003), a model-free fitting procedure developed to resolve FCS data based on a quasi-continuous distribution of highly heterogeneous diffusing components. MEMFCS was used to determine the distribution of diffusion times, which also reflects the size distribution.

### Data Analysis

The researcher performing the ThT-FCS analysis was blinded to all patient information, including diagnosis and other biomarker data until data analysis was completed. Clinical diagnosis and AT(N)-classification was completed independent of the ThT-FCS analysis results.

### Statistical Analysis

The *fSEO* variable was not normally distributed; the variable was log-transformed, and the transformed version was used for all analyses. Group differences were analyzed with ANOVA, *t*-tests, or Mann–Whitney *U* tests. Pearson product-moment correlations were performed between log(*fSEO*) and CSF Aβ_42_, T-tau, P-tau, MMSE, and CDR Sum of Boxes (CDR-SOB). The relationship between log(*fSEO*) and Aβ_42_ was further explored by multivariate linear regression. Logistic regression analysis was used to assess if log*fSEO*) could predict whether a patient belonged to the Alzheimer’s continuum. A univariate regression model with log(*fSEO*) as the only predictor was compared to two multivariate models, a clinical model including sex, age, *APOE*ε4 status, and MMSE score as covariates and a core biomarker model including CSF Aβ_42_ and P-tau as covariates. Further, we assessed whether the inclusion of log(*fSEO*) in the multivariate models improved their explanatory power. Sensitivity and specificity for the classification of A+ and A− patients were compared for all biomarkers (Aβ_42_, P-tau, T-tau, and *fSEO*) by receiver operating characteristic (ROC) curves. Optimal cut-points were determined by Youden’s index. Statistical analyses were conducted in STATA 15.1 (StataCorp, College Station, TX, USA) and R 3.4.4 (R Foundation for Statistical Computing, Vienna, Austria).

## Results

Demographic and clinical information for the 118 included patients is presented in Table 1. The amyloid groups were significantly different on all measures, except for years of education, *P* = 0.28. There is high consistency between clinical diagnosis and amyloid-positivity, 89% of patients categorized as A+ have clinical AD.

**Table 1:** Demographic and clinical characteristics for all patients, and the A− and A+ groups.

	All patients	A− group	A+ group	*P*-value
Number of participants, *n*	118	53	65	
Women, *n* (%)	61 (51.7)	22 (41.5)	39 (60.0)	0.05
Age	65.1 (8.3)	61.7 (8.9)	67.9 (6.7)	<0.001
Years of education	14.0 (3.6)	14.4 (3.4)	13.6 (3.7)	0.28
MMSE*	25.8 (4.0)	27.3 (2.9)	24.8 (4.4)	<0.001
CDR-SOB*	3.3 (2.3)	2.6 (2.3)	3.8 (2.2)	0.01
APOEε4*, *n* (%)	66 (57.4)	18 (34.6)	48 (76.2)	<0.001
Clinical diagnosis, *n* (%)				<0.001
Clinical non-AD	50 (42.4)	43 (81.1)	7 (10.8)	
Alzheimer’s clinical syndrome	68 (57.6)	10 (18.9)	58 (89.2)	
Stage, *n* (%)				<0.001
Subjective cognitive decline	11 (9.3)	9 (17.0)	2 (3.1)	
Mild cognitive impairment	48 (40.7)	31 (58.5)	17 (26.2)	
Dementia	59 (50.0)	13 (24.5)	46 (70.8)	
CSF Aβ_42_	775 (346)	1,094 (231)	516 (152)	<0.001
CSF T-tau	494 (364)	326 (167)	632 (420)	<0.001
CSF P-tau	71 (40)	53.9 (22.5)	84.2 (45.0)	<0.001
CSF *f*SEO, median (IQR)	15.6 (10.2)	14.4 (7.8)	17.4 (12.0)	0.04**
CSF log(*f*SEO)	2.78 (0.57)	2.66 (0.47)	2.88 (0.62)	0.04

Note: unless otherwise indicated, data given are mean (standard deviation) and P-values are two-tailed for t-test comparisons between A− and A+ groups. Abbreviations: Aβ, amyloid-β; APOE, apolipoprotein E; CDR-SOB, Clinical Dementia Rating Scale Sum of Boxes; CSF, cerebrospinal fluid; *f*SEO, frequency of single event occurrence; IQR, interquartile range; MMSE, Mini-Mental State Examination; n, number of patients; P-tau, phosphorylated tau; T-tau, total tau. *n = 111 for MMSE, n = 115 for CDR-SOB and APOE. **Mann–Whitney U test.

### Nanoplaque Levels and Core Biomarker Levels

**Figure 1:**
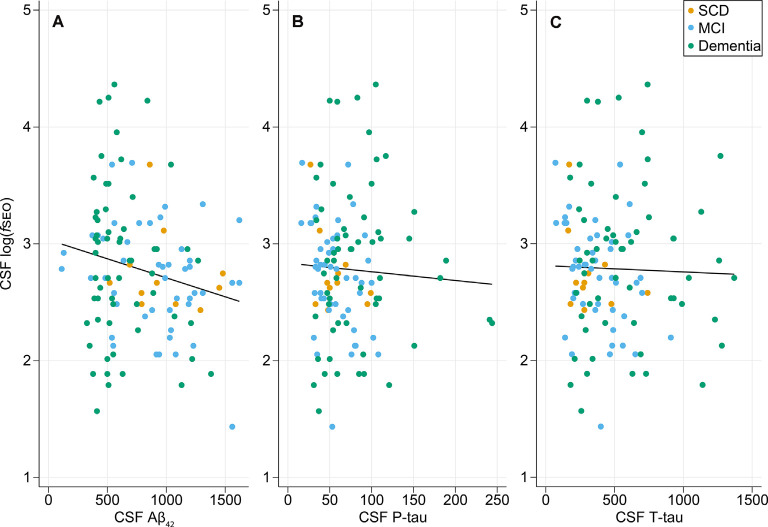
Relationship between cerebrospinal fluid (CSF) nanoplaque levels and core CSF biomarkers of AD. **(A)** Scatter plot of CSF log*f*SEO vs. CSF Aβ_42_, Pearson correlation coefficient, *r* = −0.20, *P* = 0.03. **(B)** Scatter plot of CSF log*f*SEO vs. CSF P-tau, Pearson *r* = −0.05, *P* = 0.58. **(C)** Scatter plot of CSF log*f*SEO vs. CSF T-tau, Pearson *r* = −0.03, *P* = 0.77.

The relationships between CSF log(f*SEO*) levels and the core biomarkers are presented in Figure 1. There was a weak statistically significant negative correlation between CSF Aβ_42_ and log(f*SEO*) levels, Pearson correlation coefficient *r* = −0.20, *P* = 0.03. The relationship between CSF Aβ_42_ and log(f*SEO*) remained significant when adjusting for age, APOE ε4-status and MMSE score, *β* = −135.3, *P* = 0.004.

There was no correlation between CSF log(f*SEO*) levels and T- or P-tau levels, *P* > 0.05. For the T-tau analysis, one extreme outlier with tau levels around 3,000 was excluded from the analysis. This, however, did not affect the conclusion.

### Nanoplaque Levels and Disease Severity

**Figure 2:**
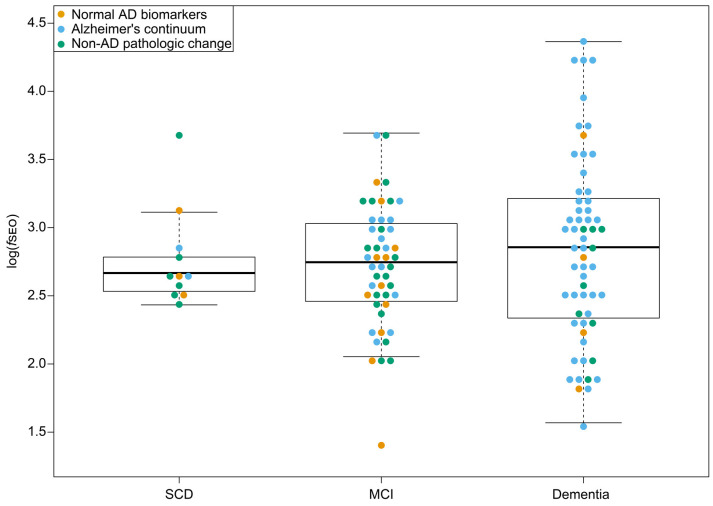
CSF nanoplaque levels according to disease stage. The box displays the first quartile (bottom line), the median (middle line), and the third quartile (top line). The upper whisker extends to the maximum data point (within 1.5 times the third quartile) and the lower whisker extends to the minimum data point (within 1.5 times the third quartile). Points beyond the whiskers represent outliers. Abbreviations: AD, Alzheimer’s disease; *f*SEO, frequency of single event occurrence; MCI, mild cognitive impairment; SCD, subjective cognitive decline.

There were no significant differences in log(f*SEO*) levels between the disease stages, *P* = 0.55, and Tukey’s *post hoc* test revealed no significant differences between neither the SCD and MCI groups, the SCD and dementia groups, nor the MCI and dementia groups, all *P* > 0.05. The distribution of log(f*SEO*) across disease stages is presented in Figure 2. CSF log(f*SEO*) levels were not associated with either MMSE-score or CDR-SOB, *P* > 0.05.

### Nanoplaque Levels Across AT(N) Classifications

**Figure 3:**
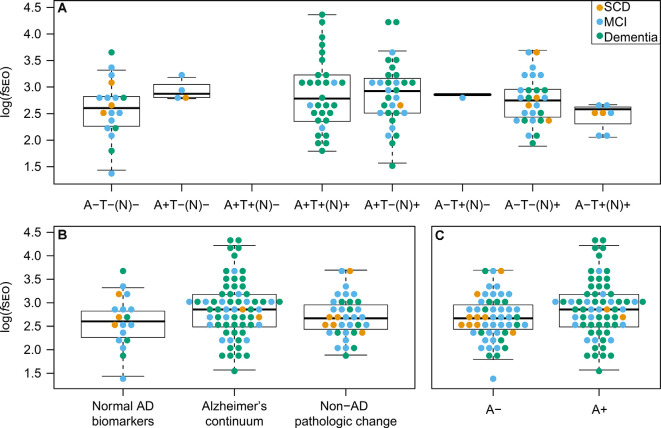
CSF nanoplaque levels according to AT(N) profile, AT(N) biomarker category, and amyloid status. **(A)** Nanoplaque levels across the eight AT(N) profiles. **(B)** Nanoplaque levels across the “Normal AD biomarkers”; “Alzheimer’s continuum” and “Non-AD pathologic change” biomarker categories. **(C)** Nanoplaque levels in A− and A+ groups, classified based on amyloid status only. The box displays the first quartile (bottom line), the median (middle line), and the third quartile (top line). The upper whisker extends to the maximum data point (within 1.5 times the third quartile) and the lower whisker extends to the minimum data point (within 1.5 times the third quartile). Points beyond the whiskers represent outliers. Abbreviations: AD, Alzheimer’s disease; *f*SEO, frequency of single event occurrence; MCI, mild cognitive impairment; SCD, subjective cognitive decline.

Nanoplaque levels across the eight AT(N)-profiles are presented in Figure 3A. Note that in this cohort there are no patients with the A + T + (N)- profile.

Nanoplaque levels were highest in patients on the Alzheimer’s continuum [log(*fSEO*) = 2.88], lower in patients with non-AD pathologic change [log(*fSEO*) = 2.69] and lowest in patients with normal AD biomarkers [log(*fSEO*) = 2.60], but this difference was not statistically significant, *P* = 0.10. The distribution across the three AT(N)-biomarker categories is presented Figure 3B.

When grouping patients by amyloid status alone, nanoplaque levels were significantly increased in A+ patients, log(*fSEO*) = 2.88 (95% confidence interval from 2.73 to 3.04) compared to A− patients, log(*fSEO*) = 2.66 (95% confidence interval from 2.53 to 2.79), *P* = 0.04. However, there was a large spread among the values, and a notable overlap between all groups, see Figure 3C.

### Classification of Patients on Alzheimer’s Continuum

The results of the logistic regressions with amyloid status as the outcome variable are presented in Table 2. In the univariate model, increased levels of nanoplaques did not significantly increase the odds of being on the Alzheimer’s continuum, *P* > 0.05. In the core biomarker model, only Aβ_42_ significantly affected the odds of being on the Alzheimer’s continuum, odds-ratio 0.22 for a 100-unit increase (95% confidence interval from 0.10 to 0.45). All variables in the clinical model were significant predictors. The odds of being on the Alzheimer’s continuum were reduced for males, odds-ratio = 0.32, and with higher MMSE-scores, odds-ratio 0.84 per point. The odds were increased for *APOE* ε4-carriers, odds-ratio = 6.12 and with increased age, odds-ratio 1.80 for 5 years (95% confidence interval from 1.27 to 2.55).

**Table 2:** Prediction of amyloid positivity (A+) by the univariate, core biomarker, and clinical models.

	OR (95% CI)	*P*-value	Explained variance, pseudo R^2^
**Univariate model**			0.03
Log(*f*SEO)	1.88 (0.92–3.82)	0.08	
**Core biomarker model**			0.81
CSF Aβ_42_	0.98 (0.98–0.99)		<0.001	
CSF P-tau	1.03 (0.99–1.07)	0.15	
**Clinical model**			0.31
Male sex	0.32 (0.12–0.86)	0.02	
Age	1.12 (1.05–1.21)	0.001	
APOEε4	6.12 (2.25–16.69)		<0.001	
MMSE	0.84 (0.73–0.96)	0.01	

*Abbreviations: Aβ, amyloid-β; APOE, apolipoproteinɛ, CI, confidence interval; CSF, cerebrospinal fluid; *f*SEO, frequency of single event occurrence; MMSE, Mini-Mental State Examination; OR, odds-ratio; P-tau, phosphorylated tau*.

When included in the core biomarker model, log(f*SEO*) remained a non-significant predictor, odds-ratio = 2.48 (95% confidence interval from 0.34 to 18.17) and did not improve the model performance, likelihood-ratio test, *χ^2^* = 0.94, *P* = 0.33. However, when adjusted for clinical variables, increased log(f*SEO*) levels significantly increased the risk of being on the Alzheimer’s continuum, odds-ratio = 4.36 (95% confidence interval from 1.44 to 13.18), *P* = 0.01. Inclusion of log(f*SEO*) increased the explained variance of the model, pseudo *R^2^* = 0.37 and improved the model performance, likelihood-ratio test, *χ^2^* = 8.04, *P* = 0.005.

**Figure 4:**
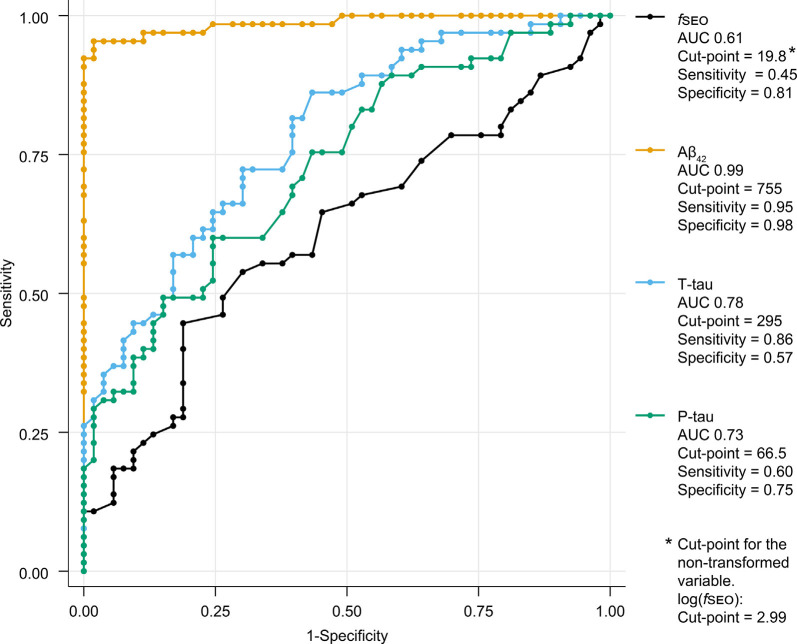
Receiver operating characteristic (ROC)-curves for classification of A+ and A− patients by CSF nanoplaque and core biomarker levels. The reported cut-point corresponds to the highest Youden’s index. Abbreviations: Aβ, amyloid-β; AUC, the area under the curve; *f*SEO, frequency of single event occurrence; P-tau, phosphorylated tau; T-tau, total tau.

The ROC-curves for the classification of patients on the Alzheimer’s continuum by CSF biomarkers Aβ_42_, T-tau, P-tau, and *f*SEO are presented in Figure 4. This figure also shows the area under the curve (AUC), cut-points, sensitivity, and specificity for each CSF marker. As expected, the CSF Aβ_42_ biomarker, based on which amyloid status was determined for most patients, had the highest sensitivity and specificity, and the largest AUC. The *f*SEO had the smallest AUC and lowest sensitivity.

### Nanoplaque Size Distributions

**Figure 5:**
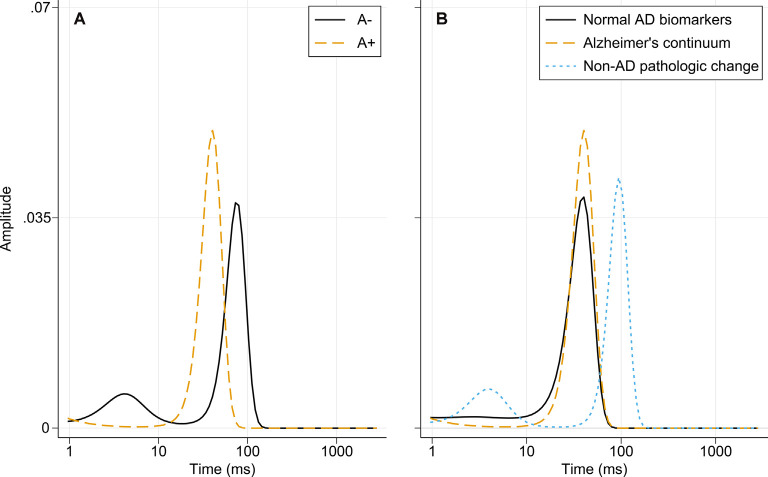
Distribution of diffusion times in different biomarker groups obtained using MEMFCS analysis of corresponding tACCs. **(A)** Diffusion times for the A− and A+ patient groups. **(B)** Diffusion times for the “Normal AD biomarkers,” “Alzheimer’s continuum” and “Non-AD pathologic change” biomarker categories. Abbreviations: AD, Alzheimer’s disease; MEMFCS, Maximum Entropy Method for Fluorescence Correlation Spectroscopy; tACC, temporal autocorrelation curves.

The diffusion times for the amyloid groups and the three biomarker categories are presented in Figure 5. The A− group has two peaks, indicating the presence of larger and smaller amyloidogenic aggregates in a dynamic equilibrium. In the A+ group, the dominant size is between the two forms in the A− group. When separating the “Normal AD biomarkers” and “Non-AD pathologic change” categories that make up the A− group, it can be seen that the nanoplaque sizes are similar for patients on the Alzheimer’s continuum and patients with normal AD biomarkers, whilst the “Non-AD pathologic change” shows the pattern seen for the A-group, with both smaller and larger nanoplaques.

## Discussion

It is hypothesized that advancing our understanding of amyloid aggregates in the AD pathogenic processes could be useful for intervention studies (Cremades and Dobson, 2018). In this study, we have investigated whether structured nanoplaques, ThT-binding amyloid aggregates enriched in secondary β-sheet structure, in CSF are related to the core biomarkers for AD. Using the recently developed ThT-FCS assay with single-molecule sensitivity, we have quantified the concentration and size of nanoplaques in the CSF. Increased levels of nanoplaques were associated with lowered CSF Aβ_42_, but not with measures of T-tau or P-tau.

The ThT-FCS assay selectively quantifies amyloidogenic aggregates with a β-sheet secondary structure (Tiiman et al., 2019). In contrast to monomeric Aβ, such aggregates are neurotoxic (Walsh et al., 1999; Ono and Tsuji, 2020); the appearance of nanoplaques could indicate pathological conversion from monomers to protofibrils. As ThT does not bind to monomers (LeVine, 1999), there is an excellent separation between the nanoplaque- and conventional Aβ_42_ measurements. While one previous study found no correlation between monomeric Aβ_42_ and amyloid oligomers in CSF (Holtta et al., 2013), we have shown a negative correlation between Aβ_42_ and nanoplaque levels. This supports the notion that the decrease in monomeric Aβ in AD CSF is partially, but not entirely, explained by its incorporation into oligomeric or fibrillar forms (Englund et al., 2009; Holtta et al., 2013). One caveat is that while ThT selectively binds aggregates with a β-sheet secondary structure, it cannot discriminate the primary structure (i.e., amino acid sequence) of the polypeptides in the nanoplaques. In addition to Aβ, several proteins, e.g., α-synuclein and prion protein, aggregate to a β-sheet structure (Soto and Pritzkow, 2018) and bind ThT (Xue et al., 2017; Cao and Yang, 2018). Hence, in theory, the detected nanoplaques could be composed of Aβ, other polypeptides, or a composite of the two (Luo et al., 2016). Moreover, Aβ aggregate folds that are not ThT-positive, i.e., that do not give rise to ThT fluorescence, may also exist.

Nanoplaque levels were increased in patients on Alzheimer’s continuum compared to patients without amyloid pathology. This is in line with previous research showing that amyloid oligomers and/or protofibrils are increased in the CSF of AD patients (Pitschke et al., 1998; Fukumoto et al., 2010; Santos et al., 2012; Holtta et al., 2013; Savage et al., 2014). However, there was substantial overlap between the biomarker groups and nanoplaque levels did not have high diagnostic accuracy for amyloid pathology. Unlike previous studies on oligomers in CSF (Fukumoto et al., 2010; Santos et al., 2012; Savage et al., 2014), nanoplaque levels were not associated with worse cognitive function in our cohort. It should be explored whether nanoplaque levels associate with changes in intra-individual cognitive performance over time. Recently, accurate plasma biomarkers such as p-tau217 have emerged, potentially revolutionizing AD diagnosis (Palmqvist et al., 2020). In the future, such markers may limit the use of CSF markers in clinical diagnosis. However, supplementary markers reflecting different aspects of AD pathology will likely continue to play an important role in furthering the understanding of AD etiology and mechanisms.

The nanoplaques identified in CSF from patients on the Alzheimer’s continuum and patients with normal AD biomarkers were similar in size and, in these groups, the size distribution was dominated by one size only. In contrast, in CSF from patients with non-AD pathologic change, both larger and smaller nanoplaques, as compared to the other groups, were observed. The implications of these findings are uncertain. While it has been shown that ThT-reactive aggregates cause toxicity in the brain by invoking neuroinflammation (De et al., 2019a,b), it is not clear whether differently sized nanoplaques have different mechanisms of toxicity. Of note, the smallest aggregates labeled by ThT contain around 40 monomers, and thus the size distribution of smaller oligomeric aggregates is not known.

One strength of the current study is the thoroughly characterized memory clinic cohort, which includes both patients on the Alzheimer’s continuum and appropriate controls with different biomarker compositions. The cognitive function of all patients has been established by several cognitive tests. All patients had at least one biomarker available in all AT(N) categories, permitting their classification according to current research criteria. A further strength is the use of the novel ThT-FCS assay, as this assay is highly suitable for biomarker studies. FCS requires small sample volumes and can measure a wide range of molecular concentrations (from around 10 pM to 100 nM; Chatterjee et al., 2017). Also, ThT-FCS assay has the utmost sensitivity and can detect single aggregated particles without relying on signal-amplification, protein separation, or immune probes. Moreover, the ThT-FCS assay can uniquely measure the size of ThT-responsive structured aggregates. The fact that the diffusion time of small structured nanoplaques (τ_D1_ = 3 ± 2 ms) is about 100 times slower than the diffusion of Rhodamine 6G (Rh6G) used for instrument calibration (τ_D,Rh6G_ = 29 ± 2 μs) and that the diffusion of the largest, not sedimented, nanoplaques (τ_D2_ = 95 ± 20 ms) is about 3,275 times slower than the diffusion of Rh6G, suggests that the size of the ThT-responsive nanoplaques floating in the CSF are about 100 nm −2 μm. Of note, the size of the nanoplaques is estimated using the Stokes-Einstein equation, $D=\frac{kT}{\text{6}\pi \eta R_{\text{H}}}$, where, *D* is the diffusion coefficient, *k* is the Boltzmann constant, *T* is the absolute temperature, η is the solvent viscosity and *R*_H_ is the hydrodynamic radius of a hypothetical compact sphere in a viscous medium, the relationship $\tau_{D}=\frac{\varpi_{XY}^{2}}{\text{4D}}$, where τ_D_ is the translational diffusion time and $\varpi_{XY}^{2}$ is the $\frac{\text{1}}{e^{\text{2}}}$ radial radius of the FCS observation volume element, and the hydrodynamic radius for Rh6G, *R*_H,Rh6G_ = 0.589 nm (Müller et al., 2008). Although this calculation applies to spherical molecules, it can be regarded here as a good enough first approximation. While the diagnostic utility of ThT-FCS does not appear to surpass that of the core biomarkers, this method could still be an important supplement to immune-based assays and contribute to a better understanding of protein aggregation and its role in AD pathology (Funke, 2011). Especially, this method could contribute to an improved understanding of conformation-dependent toxicity and the development of anti-aggregation interventions (Cremades and Dobson, 2018).

One limitation of the current study is that the intra-individual dynamics of nanoplaque levels and sizes over time cannot be inferred. Nanoplaque levels do not appear to increase linearly with disease severity or reduced levels of monomeric Aβ_42_, and the dynamics of the level and sizes of these aggregates throughout the disease process should be explored in longitudinal studies. The sample size was limited, and patients were not evenly distributed across groups; this, however, is reflective of the clinical population. Further, because the study includes a clinical population, relatively few patients with SCD were included. This is a limitation as it restricts the understanding of nanoplaque dynamics at this very early clinical stage; future studies should explore a potential role for nanoplaques at earlier disease stages.

Interestingly, ThT-reactive aggregates in CSF have recently been shown to exert toxicity by increasing neuroinflammation (De et al., 2019a). In another study, fibrillar, ThT-binding Aβ was found to increase blood-brain barrier permeability and associated inflammation *in vitro* (Parodi-Rullán et al., 2020). As such, further research should investigate the relationship between nanoplaque levels and markers of neuroinflammation. Specifically, it would be of interest to explore whether increased levels of nanoplaques predict an increase in markers of neuroinflammation. This possible link to neuroinflammation denotes potential applications of nanoplaque measurements, notably in the mechanistic understanding, diagnosis and monitoring of cerebral amyloid angiopathy-related inflammation (CAA-ri, DiFrancesco et al., 2015; Carmona-Iragui et al., 2016) and amyloid related imaging abnormalities (ARIA, Piazza and Winblad, 2016). CAA-ri is characterized by the spontaneous development of ARIA-like events, and has been put forth as a human spontaneous model of immunotherapy-induced ARIA (Piazza et al., 2013). Importantly, the concentration of CSF anti-Aβ autoantibodies is increased during the acute phases of CAA-ri (Piazza et al., 2013), and have been proposed as a promising biomarker for ARIA (Piazza and Winblad, 2016). However, the potential release of different, possibly neurotoxic, Aβ species by these autoantibodies in CAA-ri and ARIA has not been evaluated; this highlights a potential application for the ThT-FCS assay.

## Conclusion

We found that CSF nanoplaque levels were negatively correlated with monomeric Aβ_42_, but not with T- or P-tau. While CSF nanoplaque levels were increased in patients on the Alzheimer’s continuum, this method did not identify patients with amyloid pathology with the same sensitivity and specificity as the core biomarkers and did not improve the classification of patients. This method may reflect other relevant pathology, and its relationship with inflammatory markers should be explored.

## Data Availability Statement

The datasets presented in this article are not readily available because legal restrictions, imposed by the registry owners and the ethical committee, prevent us from publicly sharing the de-identified dataset due to sensitive patient information. The clinical data may be requested from the Norwegian Registry of Persons Assessed for Cognitive Symptoms at e-mail: post@aldringoghelse.no. The results of the ThT-FCS analysis are available upon reasonable request to the authors. All data availability is dependent on approval from the REC South East, contact at e-mail: post@helseforskning.etikkom.no. Requests to access the datasets should be directed to mari.aksnes@medisin.uio.no.

## Ethics Statement

The studies involving human participants were reviewed and approved by Norwegian Regional Committees for Medical and Health Research Ethics, REC South East, 2017/223. The patients/participants provided their written informed consent to participate in this study.

## Author Contributions

MA, AT, LT, NB, VV, and ABK designed the study. ABK and THE conducted the clinical assessment and diagnoses of the participants. AT did the ThT-FCS experimental and data analysis. MA did the statistical analyses and wrote the manuscript. MA, AT, NB, VV, and ABK interpreted the data. All authors read and approved the final manuscript for publication.

## Conflict of Interest

ABK and THE have worked on clinical trials for Roche (BN29553) and Boehringer-Ingelheim (1346.0023). AT, LT and VV have filed a patent application under the Patent Cooperation Treat (PCT) WO 2019/192969 A1 “Method for the Diagnosis of Amyloid-Associated Diseases.” The remaining authors declare that the research was conducted in the absence of any commercial or financial relationships that could be construed as a potential conflict of interest.
